# Supplementary material for: Bivalve Shellfish Safety in Portugal: Variability of Faecal Levels, Metal Contaminants and Marine Biotoxins during the Last Decade (2011–2020)
Source: Toxins (Basel). 2023 Jan 18;15(2):91. doi: 10.3390/toxins15020091 (PMC9962144; doi:10.3390/toxins15020091)
Supplement: Supplementary file 1 [file toxins-15-00091-s001.zip › toxins-2156073-supplementary.pdf]

Supplementary Material: Bivalve shellfish safety in Portugal: variability of faecal levels, metal contaminants and marine biotoxins during the last decade (2011-2020)

Ana Catarina Braga, Susana Margarida Rodrigues, Helena Maria Lourenço, Pedro Reis Costa and Sónia Pedro

Mensal averages of toxins concentration in the different production areas over the decade are presented in Fig. S1 and Fig. S2, for ASP and PSP, respectively.

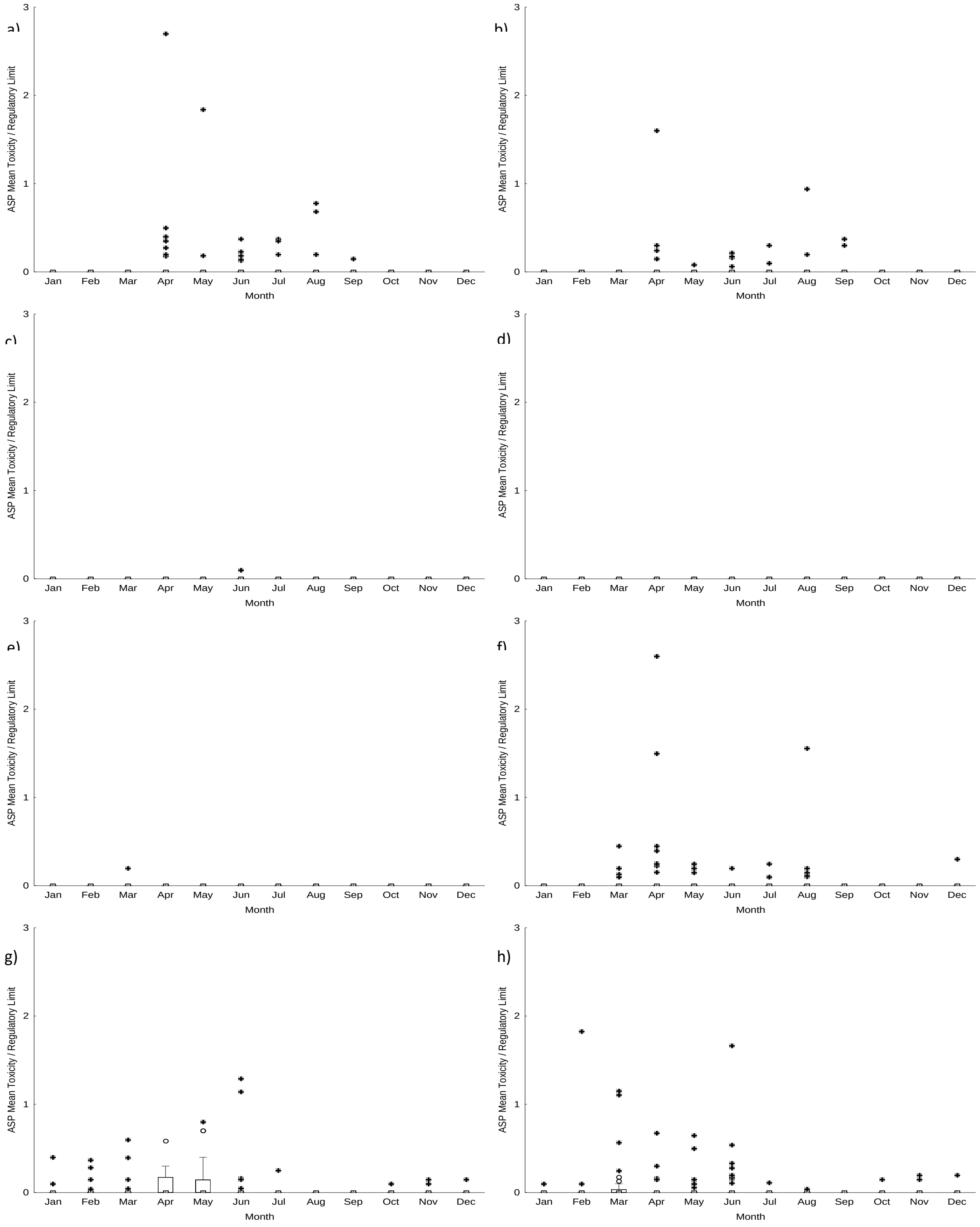

Figure S1. ASP concentrations in relation to the EU regulatory limit for mussels, between 2011 and 2020, from a) RIAV1; b) RIAV2; c) LOB; d) L5; e) L6, f) L7 and donax clams from: g) L8 and h) L9. (◘ Median; Non-Outlier Range; **o** Outliers; * Extremes).

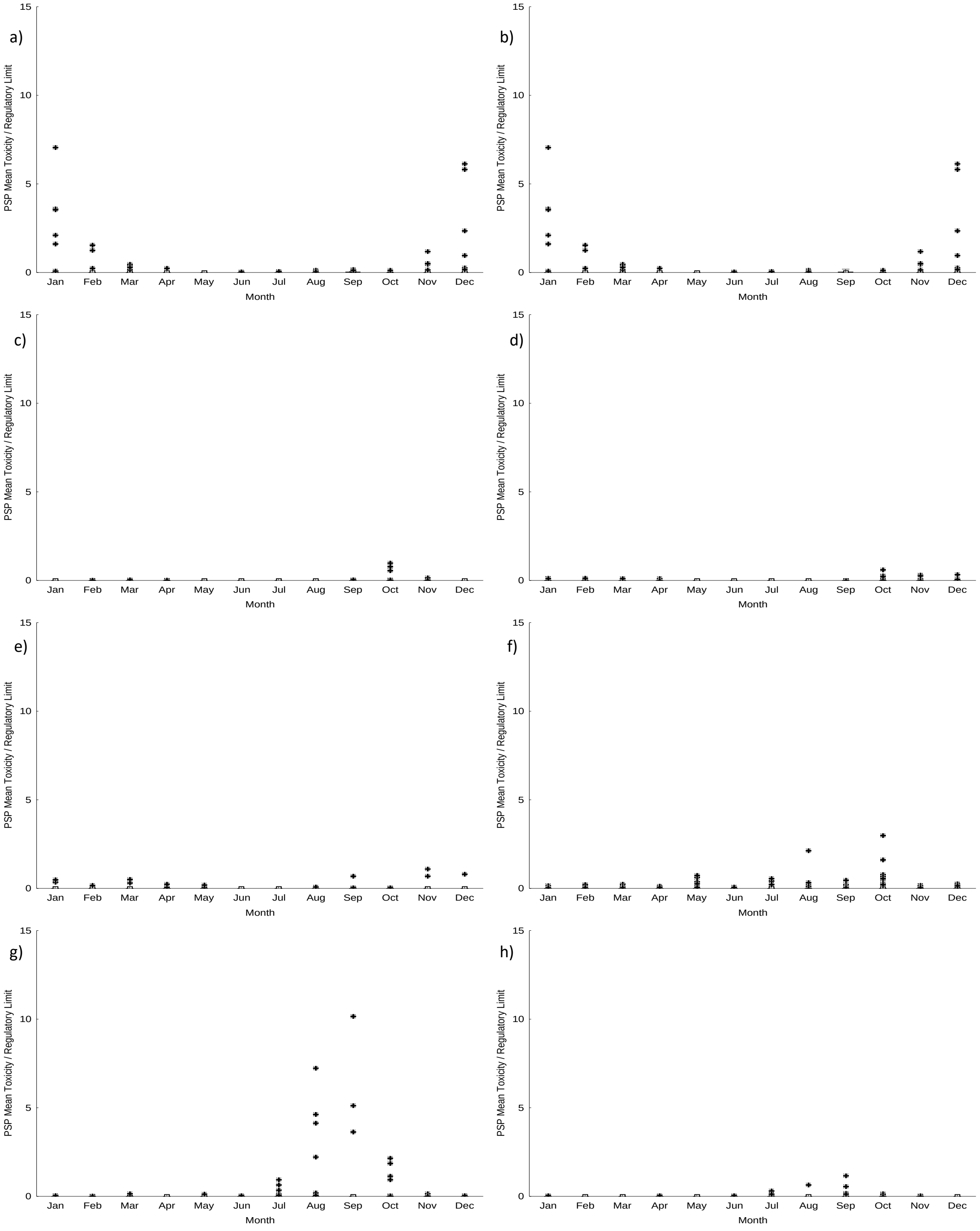

Figure S2. PSP concentrations in relation to the EU regulatory limit for mussels, between 2011 and 2020, from a) RIAV1; b) RIAV2; c) LOB; d) L5; e) L6, f) L7 and donax clams from g) L8 and h) L9. (▫ Median; Non-Outlier Range; ○ Outliers; * Extremes).
